# Impact evaluation of technical notes issued by NATJUS on healthcare judicialization

**DOI:** 10.31744/einstein_journal/2025GS0766

**Published:** 2025-05-22

**Authors:** Lucas Reis Correia, Luís Gustavo Nascimento de Paula, Julio Trecenti, Bruno Daleffi da Silva

**Affiliations:** 1 Hospital Israelita Albert Einstein São Paulo SP Brazil Hospital Israelita Albert Einstein, São Paulo, SP, Brazil.; 2 Universidade de São Paulo Faculdade de Medicina Department of Preventive Medicine São Paulo SP Brazil Department of Preventive Medicine, Faculdade de Medicina, Universidade de São Paulo, São Paulo, SP, Brazil.; 3 Terranova Consultoria São Paulo SP Brazil Terranova Consultoria, São Paulo, SP, Brazil.

**Keywords:** Technical notes, NATJUS, Health judicialization, Preliminary injunction, Impact evaluation, Costs and cost analysis, Health systems, Brazil

## Abstract

**Introduction:**

Technical Support Centers of the Judiciary (NATJUST), provide health technology assessments to judges in health-related litigations against both public and private healthcare systems. These centers provide technical notes, that can assist Brazilian Courts in making provisional rulings and determining patient access to health services during legal proceedings.

**Objective:**

This study aimed to evaluate the impact of NATJUS technical notes on preliminary injunctions in lawsuits against the Brazilian Unified Health System. Specifically, we analyzed the alignment between judicial decisions and technical notes recommendations, assessed lawsuit costs, and examined NATJUS-generated savings for Brazilian Unified Health System. Furthermore, we examined the technical notes issued by *Hospital Israelita Albert Einstein*, which manages the national NATJUS.

**Methods:**

We constructed a database of lawsuits with and without NATJUS technical notes. Using random forest models, we predicted the potential outcomes of preliminary injunctions where technical notes were available. Savings were estimated by examining the relationship between preliminary injunctions and case values, then compared with potential injunctions incorporating technical notes recommendations.

**Results:**

The technical notes recommendations and judges’ decisions were aligned in 76% of the cases, with median savings from lawsuits amounting to BRL10,120. The estimated impact of NATJUS technical notes for Brazilian Unified Health System indicated median savings of BRL1,312.74 per case per year. Between 2020 and 2022, NATJUS resulted in total savings of BRL111.4 million. Focusing on *Hospital Israelita Albert Einstein* NATJUS, the cost savings for Brazilian Unified Health System amounted to BRL27 million, which indicates a return on investment of BRL1.66 per BRL1 invested in the project.

**Conclusion:**

Our findings indicate the economic importance of technical notes in shaping preliminary injunctions, not only elevating decision quality but also fostering evidence-based judicial processes.

## In Brief

Health judicialization raises concerns regarding cost and equity in the Brazilian public health system. NATJUS provide technical notes to assist judges with preliminary injunctions, aiming for more evidence-based rulings. This study evaluates the economic impact of NATJUS, based on the premise that technical notes aid and improve rulings with public policy goals.

## Highlights

■ NATJUS technical notes yielded median savings of BRL1,312.74 per case per year.
■ Between 2020 and 2022, the notes generated BRL111.4 million in total savings for SUS.
■ HIAE NATJUS saved BRL27 million for SUS, with a return of BRL1.66 per BRL1 invested.
■ NATJUS notes help judges align their decisions with public health policy and enhance judicial outcomes.

## Graphical abstract

**Figure:**
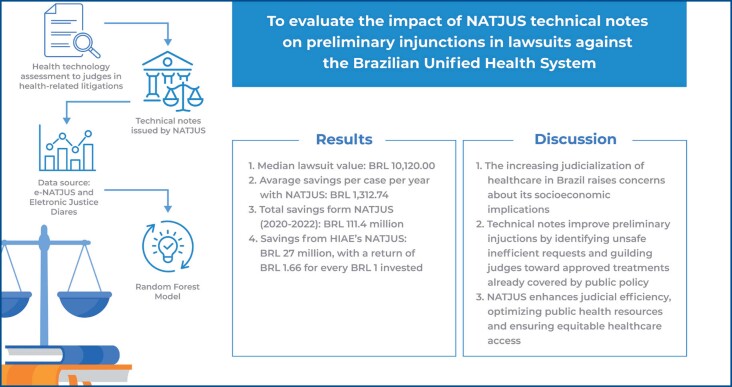

## INTRODUCTION

Judicialization of health is an increasingly common phenomenon in Brazil.^(1,2)^ It is defined by a growing number of plaintiffs seeking to resolve health-related issues through the judiciary. Lawsuits filed by patients, their families, and social organizations have addressed issues regarding access to medication, treatment, and medical procedures within both public and private healthcare systems. In the public domain, these demands are based on the Brazilian constitutional right to health, in which the judiciary acts as a guarantor when the state fails to ensure it.^(3,4)^ However, in the absence of well-established standards, this approach creates conflicts regarding the social costs of judicialization,^(5,6)^ challenging the economic rationale of efficiency^(7–9)^ and equity within the healthcare system.^(10–12)^ In limited-resource settings, these lawsuits have a significant impact on the functioning of both the Brazilian Judicial System and the Brazilian Unified Health System (SUS - *Sistema Único de Saúde*).

Diverse strategies have been conceived and implemented to address this phenomenon in the pre-procedural, procedural, and metaprocedural phases of lawsuits.^(13)^ Some of these frameworks are managed by the Brazilian National Council of Justice (CNJ - *Conselho Nacional de Justiça*), responsible for overseeing and promoting transparency within the Brazilian Judicial System.^(14,15)^ In our study, we focused on the procedural phase of the Technical Support Centers of the Judiciary (NATJUS - *Núcleos de Apoio Técnico do Poder Judiciário).* Technical Support Centers of the Judiciary provide health technology assessment to the state and federal courts in health-related litigations.^(13,16,17)^ These centers have health professionals such as physicians and pharmacists who are responsible for issuing technical notes (NTs - *Notas Técnicas*), that protect the health rights of patients during legal proceedings. These notes help judges make evidence-based rulings that determine whether patients should have access to certain healthcare services.

Much of the research on the judicialization of health relies on case studies employing qualitative methods.^(10,18,19)^ In this study, we used quantitative methods to determine the monetary value of health-related litigations, focusing on an economic analysis of the impact of NTs on preliminary injunctions in lawsuits issued against SUS. Our analyses were based on the premise that NTs can enhance the quality of court injunctions by aligning judicial claims with the best available scientific evidence. Technical notes allow the identification of requests that might result in ineffective or unsafe procedures for patients. They also help identify technologies already incorporated into public policy that can adequately meet the plaintiff's needs without resorting to unapproved treatments. Thus, NTs promote access to healthcare services that are both equitable and of high quality, aligning with public health goals and standards.

Economically, NTs can enhance the efficiency of court injunctions by reducing information asymmetry in health litigation. Previous findings suggest that judges do not always rely on scientific evidence to justify their decisions.^(10,18,20)^ Such decisions can lead to the approval of judicial claims that do not significantly benefit patients, imposing high costs on the healthcare system. Informed judgments can prevent cases rightfully favorable to the government from being won by plaintiffs, and vice versa. Thus, NT-assisted judgements rationalize the allocation of resources within SUS, leading to the secondary outcome of cost savings for the Brazilian healthcare system.

Technical Support Centers of the Judiciary operate in various forms, encompassing a national structure and several state-level units,^(1,13)^ despite ongoing efforts by the CNJ to unify the entire system.^(21)^ The national NATJUS is managed by *Hospital Israelita Albert Einstein* (HIAE) through a partnership between the Brazilian Ministry of Health and the CNJ, funded by the *Programa de Apoio ao Desenvolvimento Institucional do SUS* (PROADI-SUS).^(22)^ The current study was conducted by *Terranova Consultoria* in collaboration with the PROADI-SUS Office at HIAE.

## OBJECTIVE

To evaluate the impact of technical notes issued by NATJUS on preliminary injunctions in health-related litigations issued against Brazilian Unified Health System. Alignment between judicial decisions and technical notes recommendations was evaluated at both the national and state levels. Additionally, we compared the efficiency of *Hospital Israelita Albert Einstein* technical notes with those of other institutions. The cost of lawsuits were assessed and cost savings for Brazilian Unified Health System from using NATJUS and *Hospital Israelita Albert Einstein* technical notes were determined. Finally, we conducted a cost-benefit analysis of the *Hospital Israelita Albert Einstein* technical notes.

## METHODS

### Data collection and sample

Data were collected from two main sources—Electronic Justice Diaries (DJEs - *Diários Eletrônicos de Justiça*), and e-NATJUS.^(21,23)^ e-NATJUS is an initiative of the CNJ that was developed to serve as a national database for NATJUS NTs from various Brazilian courts. Its purpose is to centralize NT requests and issuances, facilitate data access for stakeholders (such as judges, lawyers, and physicians), and reduce conflicting judicial decisions regarding medicines and treatments in health-related litigations. It can also prevent the emergence of new health litigation that does not follow public health policies.

A database was constructed using the three-step methodology of listing, sampling, and annotation. In the first stage, we identified the total number of NTs available on the e-NATJUS platform and retrieved 37,467 data points from the HIAE database, covering approximately 52.2% of all NT-associated lawsuits on the platform. We consider this sample suitable for our study, as the remaining NTs were inaccessible owing to issues with the platform rather than with the NTs or lawsuits themselves. Our regional scope was defined by approximately 84.1% of lawsuits involving NTs from 10 Brazilian courts that were accessible (Table 1S, Supplementary Material). The DJEs provided healthcare judicialization lawsuit data from courts within our scope. Specifically, we queried terms related to the topic, extracted lawsuit numbers from surrounding pages, and cross-checked them on court websites to exclude cases beyond our scope.

At the sampling stage, we generated two random samples. One from the HIAE database, comprising lawsuits related to NTs issued by NATJUS, and the other from the DJEs database, consisting of lawsuits filed between 2015 and 2022, both before and after the creation of NATJUS, and potentially involving NTs. Finally, at the annotation stage, we applied classification forms to both sampled databases to gather data on: research scope, procedural details, NT characteristics, preliminary injunction decisions, and other rulings (Table 2S, Supplementary Material).

Our analysis relied on classified case databases comprising a total of 2,854 lawsuits filed between 2018 and 2022. After cleaning the dataset to remove inconsistencies, we retained 418 cases. These included cases without NTs or injunctions—240 from e-NATJUS (via HIAE) and 178 from DJEs. Case exclusion criteria during the annotation stage were: legal confidentiality, missing case information, and litigation not involving medications, products, or treatments (Table 3S, Supplementary Material).

**Figure 1:**
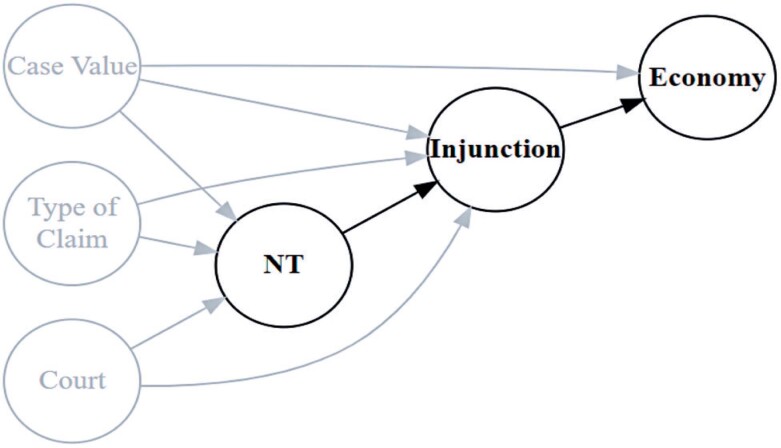
Causal framework of impact evaluation model

Notably, the monetary value of the litigation was consistently similar to the costs of medications, treatments, or medical procedures (Figure 1S, Supplementary Material). This monetary value was used to facilitate data collection.

### Model development

The model for estimating the impact of NTs on preliminary injunctions was based on the following question: in the absence of an NT, what would have been the outcome of the preliminary injunction if an NT had been available? When addressing this question, we considered whether an injunction that is initially favorable for the patient becomes unfavorable upon the inclusion of NTs, such reversals result in cost savings for SUS. Conversely, we examined whether an initially unfavorable injunction changes to a favorable one after NT inclusion, this reversal leads to financial losses for SUS. If there is no exchange of decisions, savings or losses were considered to be zero.

Figure 1 illustrates the theoretical model used in this study. The diagram suggests that the direction of the NTs (favorable/unfavorable to the patient) is influenced by the value of the litigation, type of claim (medication, treatment, or medical procedure), and the court. Similarly, the direction of the preliminary injunction (favorable/unfavorable to the patient) is influenced by the direction of the NT and the variables described earlier. Ultimately, economic impact is determined by the relationship between the injunction and monetary value of the case, when compared with the potential NT-informed injunction.

NT: technical note.

To estimate the potential impact of NTs, we fitted two models: Model 1, to predict the NT direction, and Model 2, to predict the preliminary injunction decision considering the impact of NATJUS. Both models were based on datasets containing NTs. The savings estimate was calculated by assessing the probability of a decision change. This involved applying Model 1 to determine the likelihood of a favorable NT and Model 2 to ascertain the probability of the injunction being granted. Both models were applied to a dataset that did not include NTs.

Random forest models were used to adjust both models^(24)^ because of their ability to detect complex relationships within data and ease of fit. In addition to the random forest model, logistic^(25)^ and two-stage regression^(26)^ were estimated. Furthermore, resampling techniques^(27)^ were applied to generate point and interval estimates of impact.

### Cost and economic evaluation

The cost analysis was based on point estimates and confidence intervals obtained from the random forest model. Cost savings from the use of NTs for SUS were calculated by multiplying the median estimated value by the total number of NTs identified in e-NATJUS during the 2020–2022 triennium. Similarly, cost savings from the use of HIAE NTs for SUS were calculated based on the total number of NTs identified in e-NATJUS during the same period.

We could only access the operational costs of the HIAE NATJUS, which included all direct costs, both fixed and variable, incurred by the HIAE to create and manage the NATJUS during the study period. We used this information to conduct an economic evaluation of the HIAE NTs based on the additional return on investment (ROI)^(28)^ according to the equation $\text{ROI}=\frac{Benefit\text{-}Cost}{Cost}$ . Both costs and benefits were adjusted to the actual values for 2022.

R Software (version 4.3.0) was used for all data treatments, modelling, and analyses.

## RESULTS

### Adherence of technical notes to decision-making

Table 1 presents the direction of the NT recommendations and judges’ decisions regarding preliminary injunctions, whether they were favorable to the patients or to SUS. Our findings indicated that the directions of both were aligned in 75.6% of the cases. This adherence to NTs is superior to that reported in studies that also analyzed this question, but with different methods.^(1)^

**Table 1:** Comparison between the directions of technical notes and Judges’ decisions

Direction of NTs	Direction of Judge's decisions	Volume	Agreement %
Unfavorable to patient	Unfavorable to patient	55	75.6
Favorable to patient	Favorable to patient	115	
Favorable to patient	Unfavorable to patient	17	24.4
Unfavorable to patient	Favorable to patient	38	

NTs: technical notes.

**Figure 2:**
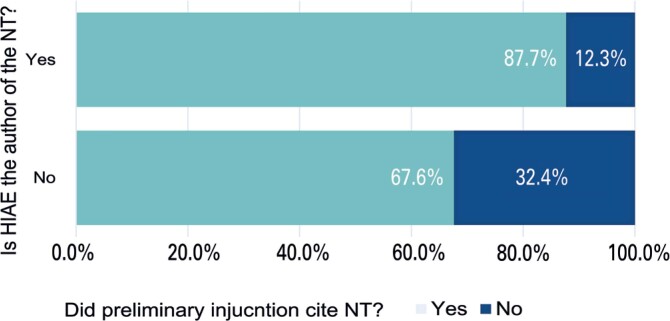
Citation of technical notes in preliminary injunctions, stratified by authorship (HIAE *versus* non-HIAE). The y-axis indicates HIAE authorship of technical notes (yes/no), while the x-axis shows whether preliminary injunctions cited technical notes (yes/no)

HIAE: *Hospital Israelita Albert Einstein*; NTs: technical notes.

*Hospital Israelita Albert Einstein* was responsible for 55% of the NTs issued by e-NATJUS during the analysis period. The decision report referenced the NTs in 79% of the cases, while HIAE NTs were mentioned in 87.7% of the cases in with they were emitted (Figure 2). The agreement of HIAE NT recommendations with the judges’ decisions increased to 82.1% (Table 2), which was higher than that of NTs from other institutions (67.6 %).

**Table 2:** Agreement between the Judge's decision and technical notes author

Author of technical note	Judge's decision	Amount	Agreement %
HIAE	Agreed	101	82.1
HIAE	Disagreed	22	17.9
Others	Agreed	69	67.6
Others	Disagreed	33	32.4

HIAE: *Hospital Israelita Albert Einstein*.

### Impact on savings

The value of litigations had a very high asymmetric distribution, as defined by the limited number of cases with exceptionally high values (Figure 2S, Supplementary Material). Therefore, instead of the mean, the median was used for analysis. The median value was BRL10,120.00, with 90% of cases exhibiting a value below BRL140,000.00, although a few cases exceeded millions.

**Figure 3:**
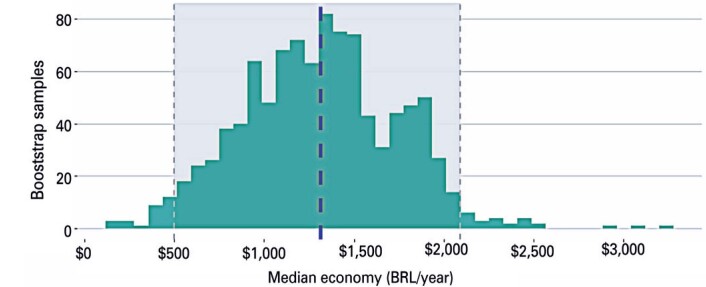
Estimated distribution of median savings

Figure 3 shows the distribution of the median savings estimates. The value highlighted with a blue dashed line is the point estimate of BRL1,312.74 per case per year. The values within the gray area denote the limits of the confidence interval estimate, between BRL498.34 and BRL2,089.24 per case per year.

BRL: Brazilian Real, the official currency of Brazil.

### Robustness

Robustness tests were conducted to validate the previous results. One test involved the use of the mean instead of the median, resulting in BRL estimates of 2,241.94. This increase was attributed to significantly higher values in a few cases. Additionally, the sequential logit and two-stage least square models estimated BRL1,190.02 and BRL1,070.81 per case per year, respectively. These values closely resembled the estimates obtained using the random forest model.

The random forest model itself was investigated to confirm the accuracy of the results. The ROC curves of Model 1 and Model 2 returned areas under the curve (AUC) of 0.991 and 0.995, respectively, on the train set (Figure 3S, Supplementary Material). Additionally, the Out of Bag prediction accuracies were found to be 77% and 80% for the respective models.

### Cost savings and cost-benefit analysis

Table 3 shows the estimated savings for SUS considering all cases identified in e-NATJUS in the 2020–2022 triennium. The point estimate indicated that the impact of NTs in preliminary injunctions resulted in cost savings of BRL111.4 million. However, we did not consider the repetition of medication, treatments, or medical procedures in the calculations, meaning that the annual cost was accounted for only once.

**Table 3:** Estimated savings considering all cases identified in e-NATJUS between 2020 and 2022

Year	Amount	CI-min (BRL)	Punctual (BRL)	CI-max (BRL)
2020	11,441	5,701,579.06	15,019,067.66	23,902,962.17
2021	38,427	19,149,950.07	50,444,691.29	80,283,115.75
2022	35,010	17,447,101.05	45,959,055.92	73,144,192.43
Total	84,878	42,298,630.18	111,422,814.87	177,330,270.34

CI-min: lower limit of confidence interval; Punctual: point estimate; CI-max: upper limit of confidence interval; BRL: Brazilian Real, the official currency of Brazil.

Considering only the NTs issued by HIAE, the point estimate indicated a cost saving for SUS amounting to BRL27,205,223.76 during the study period (Table 4). The direct cost associated with issuing these NTs was BRL10,227,301.59. Applying the ROI equation to these values, the additional ROI of HIAE NATJUS to SUS was determined to be 1.66 for every BRL1.00 invested (Table 5).

**Table 4:** Estimated savings considering HIAE cases identified in e-NATJUS between 2020 and 2022

Year	Amount	CI-min (BRL)	Punctual (BRL)	CI-max (BRL)
2020	5,169	2,575,919.46	6,785,553.06	10,799,281.56
2021	7,923	3,948,347.82	10,400,839.02	16,553,048.52
2022	7,632	3,803,330.88	10,018,831.68	15,945,079.68
Total	20,724	10,327,598.16	27,205,223.76	43,297,409.76

CI-min: lower limit of confidence interval; Punctual: point estimate; CI-max: upper limit of confidence interval; BRL: Brazilian Real, the official currency of Brazil; HIAE: Hospital Israelita Albert Einstein's.

**Table 5:** Estimated ROI of HIAE between 2020 and 2022

Year	Amount	Direct costs (BRL)	CI-min %	Punctual %	CI-max %
2020	5,169	3,540,540.93	-27.2	91.7	205.0
2021	7,923	3,168,536.18	24.6	228.3	422.4
2022	7,632	3,518,224.48	8.1	184.8	353.2
Total	20,724	10,227,301.59	1.0	166.0	323.4

CI-min: lower limit of confidence interval; Punctual: point estimate; CI-max: upper limit of confidence interval; BRL: Brazilian Real, the official currency of Brazil; HIAE: Hospital Israelita Albert Einstein's; ROI: return on investment.

## DISCUSSION

This impact evaluation demonstrated how NTs strongly influence judicial decisions in health-related litigation. According to our findings, judges aligned with NT recommendations in most cases, particularly for NTs issued by HIAE, which had higher adherence rates and were cited more often than those from other institutions. The quantitative analyses confirmed significant cost savings for SUS through NT use, with a strong ROI. While case values varied widely, median savings per case remained consistent. This supports the role of NT in balancing patient rights and sustainable healthcare spending. Model reliability was verified using multiple methods, all showing high accuracy. Together, these results highlight how evidence-based tools can improve fairness and efficiency in health judicialization.

Although our results offer valuable insights, certain limitations of our study should be noted. First, our analysis relied heavily on the HIAE database, which contained 37,467 different cases and served as the central platform for this research. However, we found that 47.8% of the NTs were inaccessible due to issues with the e-NATJUS system. Despite this limitation, we assumed that this gap did not introduce bias into the results because the inaccessibility was due to system-related issues rather than specific types of judicial cases.

The second limitation concerns the random forest model. Despite its ability to detect complex relationships within the data, it is not a quasi-experiment with counterfactual and cannot eliminate confounding variables. Additionally, the model struggles to accurately assess the uncertainty associated with predictions and tends to favor input variables with more levels or single values. One potential approach to address these issues and improve the model is to incorporate new data collections and alternative techniques for estimating uncertainties, such as Bayesian inference. Nevertheless, our results remained consistent across the different methods used, such as logistic and two-stage regression, supporting the robustness of our conclusions.

In economic theory, measuring the efficiency of the judicial system presents its own challenges. Information asymmetry appears to influence judicial decision making, as observed in settlement *versus* litigation outcomes between plaintiffs and defendants. This phenomenon aligns with Priest and Klein's divergent expectations model and Bebchuk's asymmetric information model of settlement.^(29,30)^ Technical notes can help equalize knowledge between parties and judges, enhancing both efficiency and rationality in the judicial system within the context of the judicialization of health. Moreover, according to Epstein et al.,^(31)^ information asymmetry may shape the set of motivations underlying the strategic account of judicial behavior, whether ideological, legal, or personal. Technical notes can affect the willingness of judges to make evidence-based decisions, mitigating biases that may otherwise lead to judicial outcomes that deviate from socially desirable results.

Finally, it is important to acknowledge that the resource savings discussed are based on a potential secondary outcome of the impact of NTs on preliminary injunctions. This hypothesis assumes that NTs enhance the quality of Brazilian court decisions. However, a preliminary injunction denying access to healthcare services may result in higher future healthcare costs for plaintiffs or the healthcare system if their conditions remain unresolved or worsen over time. This scenario was not considered in the present study. Additionally, we did not qualitatively assess the content of the lawsuits, NTs, or preliminary injunctions, leading to the assumption of their reasonableness. These factors introduce additional limitations to our study and require caution when interpreting the results.

Despite these limitations, NATJUS NTs allow the identification of requests that might result in inefficient or unsafe procedures for patients. Moreover, they help identify technologies already incorporated into public policy that meet the plaintiffs’ needs without resorting to unapproved treatments. As mentioned earlier, the direction of the NT recommendations and judges’ decisions were aligned in most cases, regardless of whether the decision was favorable or unfavorable to the plaintiffs. Therefore, NATJUS can promote access to healthcare services in a fair and effective manner, aligned with the proper budgetary management of health policy resources.

## CONCLUSION

Despite the limitations of the database and models used as well as the simplicity of the cost-saving hypothesis, this study provides valuable insights into the role of health technology assessment in supporting judicial decisions. The findings suggest that technical notes enhance the efficiency of court decisions by fostering a balanced and rational judicial system. They contribute to better use of public health resources while promoting access to high-quality healthcare services, aligning with Brazilian public health goals and standards. These findings pave the way for further research into the judicialization of health and functioning of NATJUS, addressing current limitations and deepening our understanding of the multifaceted issues.

## SUPPLEMENTARY MATERIAL Impact evaluation of technical notes issued by NATJUS on healthcare judicialization

Lucas Reis Correia, Luís Gustavo Nascimento de Paula, Julio Trecenti, Bruno Daleffi da Silva

**DOI: 10.31744/einstein_journal/2025GS0766**

### Table 1S List of courts and respective legal actions with technical notes included

**Table:**

Court	Volume of legal actions	Accumulated %
TRF4	8.866	23.7
TJBA	4.217	34.9
TJMS	4.051	45.7
TJRS	3.660	55.5
TJMT	3.063	63.7
TRF1	2.457	70.2
TJSP	2.105	75.9
TJPR	1.178	79.0
TJRJ	1.038	81.8
TJRN	861	84.1

### Table 2S Description of collected variables and data type

**Table:**

Variable name	Description	Data type
Base	Base classification for the record	Text
id_processo	Unique identifier for each judicial process	Text
Escopo	Scope indicator for the process	Text
info_data_dist	Process distribution date	Date
info_classe	Process class	Text
info_assunto	Subject of the process	Text
info_juiz	Judge responsible for the process	Text
info_vara	Branch of the court for the process	Text
info_comarca	Judicial district for the process	Text
tipo_acao	Type of action in the process	Text
tipo_pedido	Type of request in the process	Text
nt_relacao_liminar	Relation of the technical note to the injunction	Text
nt_codigo	Technical note code	Text
nt_autor	Author of the technical note	Text
nt_conclusao	Conclusion of the technical note	Text
nt_urgencia	Urgency status of the technical note	Text
nt_data	Date of the technical note	Date
dec_data	Date of the decision	Date
dec_cita_nt	If the decision mentions the technical note	Text
dec_liminar_result	Result of the injunction	Text
dec_motivos_discordancia	Reasons for disagreement in the decision	Text
dec_gratuidade	If the decision grants free legal aid	Text
dec_recurso	If the decision is subject to appeal	Text
dec_teve_sentenca	If the decision had a sentence	Text
dec_cita_nt_final	If the final decision mentions the technical note	Text
info_data_encerramento	Process closing date	Date
escopo_motivo_fora	Reason for exclusion from scope	Text
Obs	Additional observations	Text
valor_da_causa	Value of the case	Numeric
tempo_liminar	Duration of the injunction	Numeric
tempo_nt_liminar	Time between the technical note and the injunction	Numeric
nt_autor_hiae	If the author of the technical note is from HIAE	Text
Gratuidade	If the process has free legal aid	Text

HIAE: *Hospital Israelita Albert Einstein.*

### Table 3S Exclusion criteria during classification

**Table:**

Database	Exclusion criteria	Count	Proportion %
Base HIAE	Lawsuit under Court Secret.	40	37.0
Base HIAE	Not Found.	13	12.0
Base HIAE	It is a health judicialization lawsuit, but does not involve requests for medication, medical procedures, or products.	12	11.1
Base HIAE	Does not have a public entity as a passive party.	7	6.5
Base HIAE	Migrated lawsuit.	7	6.5
Base HIAE	Lawsuit does not have an injunction or decision.	4	3.7
Base HIAE	Not a health judicialization lawsuit.	3	2.8
Base HIAE	Physical lawsuit.	2	1.9
Base HIAE	Other criteria.	20	18.5
Base DJEs	Not a health judicialization lawsuit.	1468	71.3
Base DJEs	Physical lawsuit.	147	7.1
Base DJEs	Lawsuit under Court Secret.	127	6.2
Base DJEs	It is a health judicialization lawsuit, but does not involve requests for medication, medical procedures, or products.	114	5.5
Base DJEs	Does not have a public entity as a passive party.	56	2.7
Base DJEs	Not Found.	26	1.3
Base DJEs	Lawsuit does not have an injunction or decision.	10	0.5
Base DJEs	Not within the research time-frame.	3	0.1
Base DJEs	Other criteria.	107	5.2

## References

[1] Conselho Nacional de Justiça (CNJ), Instituto de Ensino e Pesquisa (Insper) (2019). Judicialização da saúde no Brasil: perfil das demandas, causas e propostas de solução.

[2] Conselho Nacional de Justiça (CNJ) (2021). Judicialização e saúde: ações para acesso à saúde pública de qualidade.

[3] Wang D (2009). Poder Judiciário e políticas públicas de saúde: participação democrática e equidade. Cad Gestão Pública Cidadania.

[4] Vasconcelos NP (2020). Solução do problema ou problema da solução? STF, CNJ e a judicialização da saúde. Rev Estudos Institucionais.

[5] Tribunal de Contas de União (TCU) (2017). Relatório de Auditoria: Acórdão 1.787/2017 – TCU – Plenário.

[6] Vieira FS (2018). Evolução do gasto com medicamentos do Sistema Único de Saúde no período de 2010 a 2016. Texto para discussão do IPEA n. 2356.

[7] Van Staveren I, Peil J, Staveren IV (2009). Handbook of Economics and Ethics.

[8] Ferraz OL, Vieira FS (2009). Direito à saúde, recursos escassos e equidade: os riscos da interpretação judicial dominante. Dados.

[9] Vieira FS (2023). Judicialization and right to health in Brazil: a trajectory of matches and mismatches. Rev Saude Publica.

[10] Andia TS, Lamprea E (2019). Is the judicialization of health care bad for equity? A scoping review. Inter J Equity Health.

[11] Boarati V, Diaz MD (2020). Fatores Associados a Judicialização por Portadores de DM e Perfil dos Demandantes da Justiça Gratuita.

[12] Lopes LM, Acurcio FA, Diniz SD, Coelho TL, Andrade EG (2019). (Un)Equitable distribution of health resources and the judicialization of healthcare: 10 years of experience in Brazil. Inter J Equity in Health.

[13] Vasconcelos NP (2021). Between justice and public management: interinstitutional collaboration in health litigation. Rev Adm Pública.

[14] Conselho Nacional de Justiça (CNJ) (2010). Recomendação n. 31, de 30 de março de 2010. Recomenda aos Tribunais a adoção de medidas visando melhor subsidiar os magistrados e demais operadores do direito, para assegurar maior eficiência na solução das demandas judiciais envolvendo a assistência à saúde.

[15] Conselho Nacional de Justiça (CNJ) (2010). Resolução n. 107, de 06 de abril de 2010. Institui o Fórum do Judiciário para monitoramento e resolução das demandas de assistência à saúde.

[16] Conselho Nacional de Justiça (CNJ) (2016). Resolução n. 238, de 06 de novembro de 2016. Dispõe sobre a criação e manutenção, pelos Tribunais de Justiça e Regionais Federais de Comitês Estaduais da Saúde, bem como a especialização de vara em comarcas com mais de uma vara de fazenda Pública.

[17] Yamauti SM, Barreto JO, Barberato-Filho S, Lopes LC (2020). Strategies implemented by public institutions to approach the judicialization of health care in Brazil: a systematic scoping review. Frontiers Pharmacol.

[18] Lyra PF, Araújo DC, Santos GA, Sondré-Alves BM, Santos de Jesus EM, Lyra DP (2021). The quality of research on judicialization and its influence on public policies on access to medicines in Brazil: A systematic review. Cien Saude Coletiva.

[19] Oliveira MR, Delduque MC, Souza MF, Mendonça AV (2015). Judicialização da saúde: para onde caminham as produções científicas?. Saúde Debate.

[20] Dias ER, Silva GB (2016). Evidence-Based Medicine in judicial decisions concerning right to healthcare. einstein (São Paulo).

[21] Conselho Nacional de Justiça (CNJ) (2016). Provimento n. 84, de 14 de agosto de 2019. Dispõe sobre o uso e o funcionamento do Sistema Nacional de Pareceres e Notas Técnicas (e-Natjus).

[22] Brasil. Presidência da República (2021). Lei Complementar n. 187, de 16 de dezembro de 2021. Dispõe sobre a certificação das entidades beneficentes e regula os procedimentos referentes à imunidade de contribuições à seguridade social.

[23] Conselho Nacional de Justiça (CNJ) Sistema e-NATJUS - Portal CNJ.

[24] Breiman L (2001). Random forests. Machine Learning.

[25] Nelder JA, Wedderburn R (1972). Generalized linear models. J Royal Statistical Society: Series A (General).

[26] Angrist JD, Imbens GW (1995). Two-stage least squares estimation of average causal effects in models with variable treatment intensity. J Am Statistical Association.

[27] Davison AC, Hinkley DV (1997). Bootstrap methods and their application (No. 1).

[28] Brasil. Ministério da Saúde (2014). Diretrizes Metodológicas: Diretriz de Avaliação Econômica.

[29] Lee YH, Klerman D (2016). The Priest-Klein hypotheses: Proofs and generality. Intern Review Law Economics.

[30] Waldfogel J (1998). Reconciling asymmetric information and divergent expectations theories of litigation. J Law Economics.

[31] Epstein L, Knight J (2013). Reconsidering judicial preferences. Annual Review Political Science.

